# Complete mitochondrial genome of the jellyfish, *Rhopilema esculentum* Kishinouye 1891 (Cnidaria: Scyphozoa) and the phylogenetic relationship in the related species

**DOI:** 10.1080/23802359.2017.1303342

**Published:** 2017-03-23

**Authors:** Yantao Wang, Song Sun

**Affiliations:** a Key Laboratory of Marine Ecology and Environmental Sciences, Institute of Oceanology, Chinese Academy of Sciences, Qingdao, China;; b Laboratory for Marine Ecology and Environmental Science, Qingdao National Laboratory for Marine Science and Technology, Qingdao, China;; c Jiaozhou Bay Marine Ecosystem Research Station, Institute of Oceanology, Chinese Academy of Sciences, Qingdao, China

**Keywords:** *Rhopilema esculentum*, mitochondrial genome, next generation sequencing, phylogenetic relationship

## Abstract

The complete mitochondrial genome of *Rhopilema esculentum* collected from Jiaozhou Bay, China, was determined by next-generation sequencing. The mitogenome is a circular molecule 15,855 bp in length, including 13 protein-coding genes (Cox 1, Cox2, Atp 8, Atp 6, Cox 3, ND2, ND5, ND 6, ND3, ND4L, ND1,ND4, Cytb), 5 tRNAs (tRNA-Trp, tRNA-Met, tRNA-Leu, tRNA-Ser, tRNA-Gln), 2 rRNA genes (small subunit RNA and large subunit RNA) and 1 putative control region. The phylogenetic tree in the related species showed that *R. esculentum* is close to *Nemopilema nomurai*.

*Rhopilema esculentum* Kishinouye 1891 (Cnidaria: Scyphozoa: Rhizostomeae: Rhizostomatidae) inhabits a wide range of the northwestern Pacific region including seas around Japan, Korea, and China. In China, *R. esculentum* is widely distributed in the Bohai Sea, the Yellow Sea, the East China Sea, and the northern South China Sea (Dai et al. 2004; Jiang et al. 2007). The edible jellyfish *R. esculentum* has been exploited in Chinese waters for more than 1700 years and is one of the most abundant fishery species in China (Omori & Nakano 2001).

To date, a few reports are available on complete mitochondrial genomes as in *Aurelia aurita* (Shao et al. 2006), *Aurelia* sp. nov. (Hwang et al. 2014), *Craspedacusta sowerbyi* (Zou et al. 2012), and *Chrysaora quinquecirrha* (Hwang 2014). It would be therefore important to analyze the complete mitochondrial genome to better understand the molecular phylogenetic relationship between Cnidaria species. In the present paper, we first report the complete mitochondrial DNA from *N. nomurai* in order to obtain basic genetic information within the genus Rhizostomatidae. It is expected that the information obtained from complete mitochondrial genome sequence of *R. esculentum* would provide a useful genetic resource to be utilized in the future investigation on population genetics and phylogenomics of Scyphozoa. DNA from about 1 g bell tissue of a single specimen of *R. esculentum* weighing ∼5 kg collected from Jiaozhou Bay, China, (36.10N, 120.30E) was extracted by the standard phenol-chloroform extraction method (Sambrook & Russell 2001) and part of the rest of the sample was preserved in −20 °C at KLMEES Institute of Oceanology with the voucher no. Rmit001.

The complete mitogenome of *R. esculentum* is 15,855 bp in length and the GenBank accession No. is KY454768. It consists of 13 protein-coding genes (including Cox1, Cox2, Atp8, Atp6, Cox3, ND2, ND5, ND 6, ND3, ND4L, ND1, ND4, Cytb), 5 tRNAs (tRNA-Trp, tRNA-Met, tRNA-Leu, tRNA-Ser, tRNA-Gln), 2 rRNA genes(small subunit RNA and large subunit RNA), and 1 putative control region. All the genes showed complete stop codons using TAA and TAG. There is also slight anti-G bias (13.70% and 13.24%) on the 2nd and 3rd position of all the genes. The start codon of ND3 and ND6 are different from other species, such as *A. aurita* (Shao et al. 2006).

The mitochondrial genome base composition for 13 genes was 28.12% for A, 37.01% for T, 17.40% for G and 17.37% for C. The A + T base composition (65.13%) was higher than G + C(34.77%) based on the sequences of 13 genes, suggesting that the *R. esculentum* has low G + C ratio in the mitochondrial genome. Phylogenetic relationship revealed NJ tree among 5 related species based on the complete mitochondrial genome downloaded from NCBI (Figure 1). The NJ phylogenetic tree showed that the *R. esculentum* is close to *Nemopilema nomurai* (GenBank No. KY454767) clustered in a separate branch, and was far related to other species.

**Figure 1.**
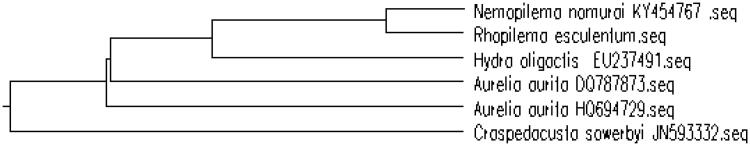
Phylogenetic relationship revealed by NJ tree.
